# Regulatory Effects of Endometriosis-Associated Genetic Variants: A Multi-Tissue eQTL Analysis

**DOI:** 10.3390/diseases13080248

**Published:** 2025-08-06

**Authors:** Asbiel Felipe Garibaldi-Ríos, Perla Graciela Rodríguez-Gutiérrez, Jesús Magdiel García-Díaz, Guillermo Moisés Zúñiga-González, Luis E. Figuera, Belinda Claudia Gómez-Meda, Ana María Puebla-Pérez, Ingrid Patricia Dávalos-Rodríguez, Blanca Miriam Torres-Mendoza, Itzae Adonai Gutiérrez-Hurtado, Martha Patricia Gallegos-Arreola

**Affiliations:** 1 División de Genética, Centro de Investigación Biomédica de Occidente (CIBO), Centro Médico Nacional de Occidente (CMNO), Instituto Mexicano del Seguro Social (IMSS), Guadalajara 44340, Jalisco, Mexico; asbiel.garibaldi4757@alumnos.udg.mx (A.F.G.-R.); pergra.pgrg@gmail.com (P.G.R.-G.); luisfiguera@yahoo.com (L.E.F.); ingriddavalos@hotmail.com (I.P.D.-R.); 2 Doctorado en Genética Humana, Centro Universitario de Ciencias de la Salud (CUCS), Universidad de Guadalajara (UdeG), Guadalajara 44340, Jalisco, Mexico; 3 Unidad de Biotecnología Médica y Farmacéutica, Centro de Investigación y Asistencia en Tecnología y Diseño del Estado de Jalisco, Guadalajara 44270, Jalisco, Mexico; jegarcia_al@ciatej.edu.mx; 4 División de Medicina Molecular, Centro de Investigación Biomédica de Occidente (CIBO), Centro Médico Nacional de Occidente (CMNO), Instituto Mexicano del Seguro Social (IMSS), Sierra Mojada 800, Col. Independencia, Guadalajara 44340, Jalisco, Mexico; mutagenesis95@hotmail.com; 5 Departamento de Biología Molecular y Genómica, Instituto de Genética Humana “Dr. Enrique Corona Rivera”, Centro Universitario de Ciencias de la Salud (CUCS), Universidad de Guadalajara (UdeG), Guadalajara 44340, Jalisco, Mexico; belinda.gomez@academicos.udg.mx (B.C.G.-M.); itzae.gutierrez@academicos.udg.mx (I.A.G.-H.); 6 Laboratorio de Inmunofarmacología, Centro Universitario de Ciencias Exactas e Ingenierías, Universidad de Guadalajara (UdeG), Guadalajara 44430, Jalisco, Mexico; ana.puebla@academicos.udg.mx; 7 Laboratorio de Inmunodeficiencias y Retrovirus Humanos, División de Neurociencias, Centro de Investigación Biomédica de Occidente (CIBO), Instituto Mexicano del Seguro Social (IMSS), Guadalajara 44340, Jalisco, Mexico; blanca.torresm@imss.gob.mx; 8 Departamento de Clínicas Médicas, Centro Universitario de Ciencias de la Salud (CUCS), Universidad de Guadalajara (UdeG), Guadalajara 44340, Jalisco, Mexico

**Keywords:** endometriosis, gene expression regulation, genetic variants, expression quantitative trait loci, tissue distribution, genetics of endometriosis

## Abstract

Backgroud. Endometriosis is a chronic, estrogen-dependent inflammatory disease characterized by the ectopic presence of endometrial-like tissue. Although genome-wide association studies (GWAS) have identified susceptibility variants, their tissue-specific regulatory impact remains poorly understood. Objective. To functionally characterize endometriosis-associated variants by exploring their regulatory effects as expression quantitative trait loci (eQTLs) across six physiologically relevant tissues: peripheral blood, sigmoid colon, ileum, ovary, uterus, and vagina. Methods. GWAS-identified variants were cross-referenced with tissue-specific eQTL data from the GTEx v8 database. We prioritized genes either frequently regulated by eQTLs or showing the strongest regulatory effects (based on slope values, which indicate the direction and magnitude of the effect on gene expression). Functional interpretation was performed using MSigDB Hallmark gene sets and Cancer Hallmarks gene collections. Results. A tissue specificity was observed in the regulatory profiles of eQTL-associated genes. In the colon, ileum, and peripheral blood, immune and epithelial signaling genes predominated. In contrast, reproductive tissues showed the enrichment of genes involved in hormonal response, tissue remodeling, and adhesion. Key regulators such as *MICB*, *CLDN23*, and *GATA4* were consistently linked to hallmark pathways, including immune evasion, angiogenesis, and proliferative signaling. Notably, a substantial subset of regulated genes was not associated with any known pathway, indicating potential novel regulatory mechanisms. Conclusions. This integrative approach highlights the com-plexity of tissue-specific gene regulation mediated by endometriosis-associated variants. Our findings provide a functional framework to prioritize candidate genes and support new mechanistic hypotheses for the molecular pathophysiology of endometriosis.

## 1. Introduction

Endometriosis is a chronic, estrogen-dependent inflammatory disease characterized by the presence of endometrial-like epithelium and/or stroma outside the uterine cavity [1]. These ectopic lesions undergo cyclical hormonal stimulation, leading to local bleeding, chronic pelvic inflammation, fibrosis, and the formation of adhesions [2]. Clinically, endometriosis presents a wide spectrum of severity, ranging from superficial peritoneal implants to deep infiltrating disease, commonly affecting pelvic organs such as the ovary, vagina [3,4,5], and in rare cases on intestinal segments like the colon sigmoid and ileum [6,7]. This condition affects approximately 10% of women of reproductive age, corresponding to over 190 million women worldwide. It is identified in 40–50% of women and adolescents with chronic pelvic pain and in 30–40% of those experiencing infertility. However, the true prevalence remains uncertain due to diagnostic challenges, as confirmation still relies on histological examination of surgically obtained tissue. Delays in diagnosis, underreporting, and the absence of non-invasive biomarkers contribute to this underestimation [1,3].

Despite extensive research, the pathogenesis of endometriosis remains incompletely understood. The most widely accepted theory is retrograde menstruation; whereby endometrial fragments reflux through the fallopian tubes into the peritoneal cavity during menstruation. Yet, since this phenomenon occurs in up to 90% of women, additional factors such as genetic susceptibility, aberrant immune surveillance, localized estrogen production, inflammation, and epigenetic modifications have been proposed to play critical roles. Alternative theories include coelomic metaplasia, the persistence of embryonic cell rests, lymph vascular metastasis, and stem cell origins, especially in cases of extra-pelvic endometriosis [1,2].

Multiple risk factors have been associated with the development of endometriosis, including low birth weight, early menarche, short menstrual cycles, nulliparity, and environmental exposures such as endocrine-disrupting chemicals (e.g., phthalates, perfluorochemicals), which may alter hormonal and immune homeostasis [3,8]. However, these factors alone do not fully explain the complex and heterogeneous nature of the disease. Genetic susceptibility is also believed to play a key role, with increasing evidence pointing to inherited variants that influence immune, hormonal, and cellular processes relevant to endometriosis development [9].

In the era of genomic medicine, understanding how genetic variants shape the molecular landscape of endometriosis represents a critical step toward unraveling its pathogenesis. While genome-wide association studies (GWAS) have identified multiple loci associated with increased disease risk [9,10], most of these variants reside in non-coding regions [9], complicating the interpretation of their functional significance. To address this, integrating GWAS findings with expression quantitative trait loci (eQTL) data offers a powerful strategy to elucidate how genetic variation modulates gene expression in a tissue-specific manner.

In the present study, we explored the regulatory impact of endometriosis-associated genetic variants across six relevant human tissues: uterus, ovary, vagina, colon, ileum, and peripheral blood. These tissues were selected based on their direct involvement in lesion development (reproductive and intestinal tissues) or their utility in capturing systemic immune and inflammatory signals (blood). Although GTEx [11] data represent gene expression in healthy tissues, this approach enables the identification of baseline regulatory effects that may predispose certain individuals to disease even before pathological changes occur. To this end, we first curated a list of genetic variants previously associated with endometriosis from the GWAS Catalog [12], and then systematically examined whether these variants act as eQTLs, modifying gene expression in each selected tissue using GTEx data. By uncovering these constitutive regulatory patterns, our work provides insights into early molecular disruptions potentially involved in the development of endometriosis.

## 2. Methodology

### 2.1. Variant Selection and Functional Annotation

A total of 710 genome-wide significant genetic associations for endometriosis were retrieved from the GWAS Catalog [12] (https://www.ebi.ac.uk/gwas/. Accessed on 12 May 2025) using the ontology identifier EFO_0001065. Only variants with a *p*-value less than 5 × 10^−8^ were included. Variants without a standardized identifier (rsID) were excluded, resulting in 556 entries with valid rsIDs. In cases where a variant appeared multiple times across different studies or gene associations, only the entry with the lowest *p*-value was retained. The final dataset comprised 465 unique variants used for downstream analysis. The variants identified were functionally annotated using the Ensembl Variant Effect Predictor (VEP) [13] (https://www.ensembl.org/. Accessed on 13 May 2025). This tool was used to determine the genomic location of each variant (e.g., intronic, exonic, intergenic, or UTR), the associated gene, chromosome, and the functional region of the genome where the variant was located.

### 2.2. eQTL Identification in Relevant Tissues

The selected variants were cross-referenced with tissue-specific eQTL datasets from the GTEx v8 database [11] (https://gtexportal.org/home/. Accessed on 13 May 2025). Tissues with biological relevance to the pathophysiology of endometriosis were included, encompassing both reproductive and digestive systems. Specifically, the uterus, ovary, vagina, sigmoid colon, ileum, and peripheral blood (whole blood) were analyzed. Only significant eQTLs were retained, defined as those with an adjusted *p*-value (false discovery rate, FDR) below 0.05. For each variant, the regulated gene, the slope (representing the direction and magnitude of the regulatory effect), the adjusted *p*-value, and the corresponding tissue were documented. The slope represents the normalized effect size, which estimates how gene expression changes for each additional copy of the alternative allele. For example, a slope of +1.0 indicates a twofold increase in expression, while −1.0 reflects a 50% decrease. Even moderate values, such as ±0.5, may represent meaningful regulatory effects in disease-relevant genes [11].

### 2.3. Functional Analysis Using Hallmark Gene Sets

To explore the biological functions potentially associated with the regulated genes, a functional analysis was performed using the Cancer Hallmarks platform [14] (https://www.cancerhallmarks.com, Accessed on 18 May 2025). For each tissue, two gene sets were selected: (1) the top 10 genes regulated by the highest number of eQTL variants and (2) the genes with the highest average slope values. Gene lists for each of the six analyzed tissues (sigmoid colon, ileum, ovary, uterus, vagina, and whole blood) were manually submitted to Cancer Hallmarks platform and compared against two curated reference collections: the MSigDB Hallmark Gene Sets, from the Molecular Signatures Database and the Cancer Hallmark Gene Set. Results were downloaded in tabular format and reviewed to identify hallmark categories in each gene set. Genes that did not match any category were classified as “Not linked to Hallmark.”

### 2.4. Statistical Considerations

To ensure analytical robustness, we included only genetic variants with genome-wide significance (*p* < 5 × 10^−8^) as reported in the GWAS Catalog [12]. For downstream analyses, we considered only significant eQTLs from GTEx [11] that passed false discovery rate correction (FDR < 0.05). The slope values provided by GTEx [11] were used to assess both the direction and magnitude of the regulatory effect. Genes were prioritized based on two main criteria: the number of associated variants and their average slope value across tissues.

## 3. Results

### 3.1. Characterization of Endometriosis-Associated GWAS Variants

**Figure 1:**
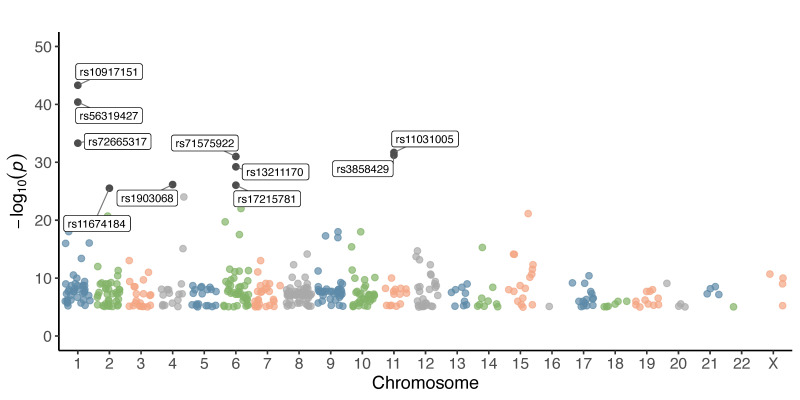
Chromosomal distribution and significance of GWAS variants associated with endometriosis. Each dot represents a single variant plotted according to its chromosomal position (x-axis) and –log_10_(*p*-value) (y-axis). Different colors are used to distinguish chromosomes.

We began the analysis with the 465 endometriosis-associated variants retained after filtering, as described in the Section 2. These variants were distributed across all 22 autosomal chromosomes and the X chromosome. Chromosome 8 harbored the highest number of variants (*n* = 66), followed by chromosomes 6 (*n* = 43), 1 (*n* = 42), 2 (*n* = 38), 9 (*n* = 37), and 10 (*n* = 33). In contrast, chromosomes 16 and 22 contained only one variant each. A total of four variants were located on the X chromosome (Figure 1, Table 1). The ten most significant variants presented *p*-values ranging from 5 × 10^−44^ to 3 × 10^−26^. Four of these were located on chromosome 1: rs10917151 (*p* = 5 × 10^−44^), rs56319427 (*p* = 4 × 10^−41^), rs72665317 (*p* = 5 × 10^−34^), and rs11674184 (*p* = 3 × 10^−26^). Three variants were found on chromosome 6: rs71575922 (*p* = 1 × 10^−31^), rs13211170 (*p* = 6 × 10^−30^), and rs17215781 (*p* = 9 × 10^−27^). Variants rs11031005 (*p* = 2 × 10^−32^) and rs3858429 (*p* = 6 × 10^−32^) were located on chromosome 11, while rs1903068 (*p* = 7 × 10^−27^) was identified on chromosome 4 (Figure 1, Table 1).

### 3.2. Functional Annotation and Gene Distribution of GWAS Variants

**Figure 2:**
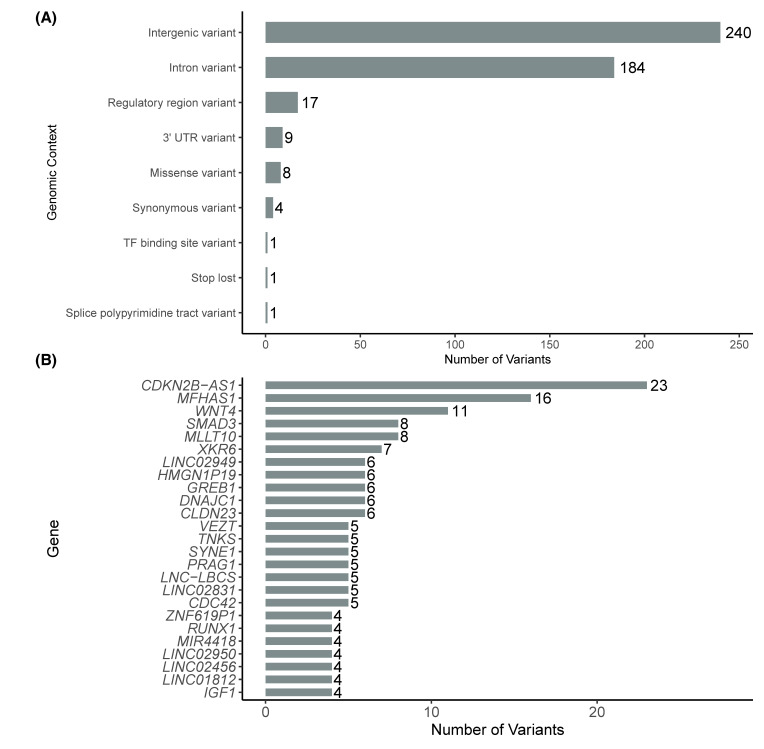
Distribution of GWAS variants by genomic context (**A**) and by number of variants in the top 20 genes with the highest number of associated variants (**B**).

When analyzing the functional annotation of the variants, we observed that most were in intergenic regions (*n* = 240) and intronic regions (*n* = 184). A smaller number of variants were found in regulatory regions (*n* = 17), within the 3′ untranslated region (3′ UTR, *n* = 9) and as missense variants (*n* = 8). Additionally, a limited number of synonymous variants were identified (*n* = 4), along with one variant in each of the following categories: transcription factor binding site, stop lost variant, and polypyrimidine tract involved in splicing (Figure 2A).

We also observed that some genes accumulated a higher number of associated variants. The top 20 genes with the highest counts included *CDKN2B-AS1* (*n* = 23); *MFHAS1* (*n* = 16); *WNT4* (*n* = 11); *SMAD3* and *MLLT10* (*n* = 8); *CDC42* and *SKIDA1* (*n* = 7); and *IL1A*, *FSHB*, and *RAB5B* (*n* = 6). The top 10 genes with more GWAS-variants are shown in Figure 2B.

### 3.3. Identification of eQTL-Regulated Genes in Endometriosis-Associated Tissues

**Figure 3:**
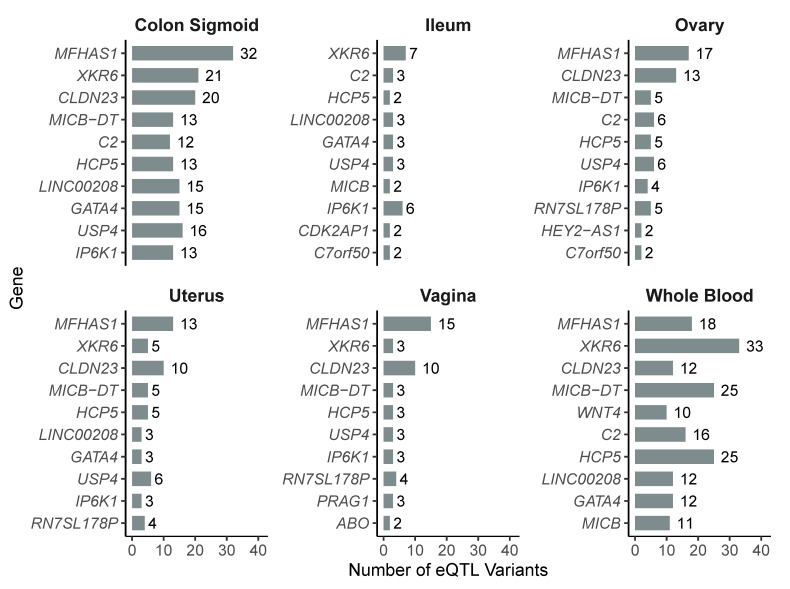
Top 10 most frequently regulated genes by eQTL variants in each of the six analyzed tissues.

To characterize the most frequently affected genes, we identified the ten genes with the highest number of associated eQTL variants in each tissue. In whole blood, *XKR6* was the most regulated gene (33 variants), followed by *MICB-DT* and *HCP5* (25 variants each), and *MFHAS1* (18). In the sigmoid colon, *MFHAS1* (32 variants), *XKR6* (21), and *CLDN23* (20) were the top-ranked genes. In the ovary, *MFHAS1* (17), *CLDN23* (13), and *USP4* (6) showed the highest regulatory counts. In the uterus, *MFHAS1* (13), *CLDN23* (10), and *XKR6* (5) were the most affected. In the vagina, the top regulated genes included *MFHAS1* (15), *CLDN23* (10), and *MICB-DT* (4). Lastly, in the ileum, *XKR6* (7), *IP6K1* (6), and a group of genes with three variants each (*LINC00208*, *GATA4*, *USP4*) were the most frequent (Figure 3).

**Figure 4:**
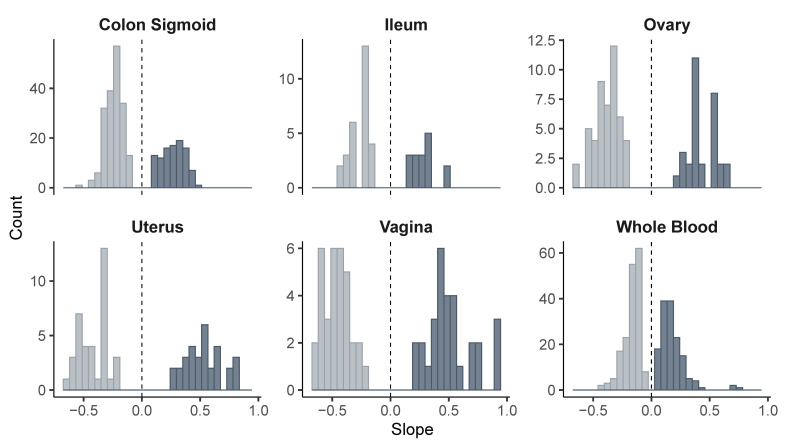
Distribution of slope values from eQTL variants across tissues. Light and dark colors indicate negative and positive slopes, respectively, to visually distinguish the direction of the regulatory effect.

We then evaluated the overall distribution of the regulatory effects by analyzing the slope values in each tissue. In all cases, both positive and negative effects on gene expression were observed. In the sigmoid colon, a bimodal distribution of slope values was evident, while in whole blood, effects were concentrated near zero. In the ileum, ovary, uterus, and vagina, the distributions were broader, with variants associated with both increased and decreased gene expression (Figure 4).

Next, we calculated the average slope values per gene and tissue to identify those with the strongest regulatory effects. In the vagina, *MICB, MICB-DT*, and *HCP5* had the highest slope values (≥0.8), regulated by variants rs2534685, rs2534687, and rs2894221, respectively. In the uterus, these same genes showed slopes around 0.62, while *GATA4* and *LINC00208*, regulated by rs13248109, rs13250871, and rs4840573, displayed more pronounced negative effects (slope ≈ −0.51).

**Figure 5:**
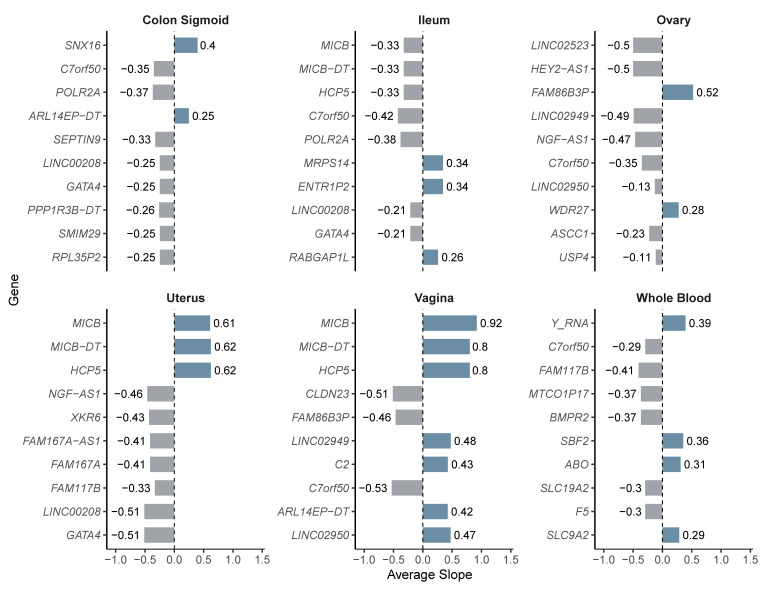
Average slope values per gene and tissue for eQTL-regulated genes. Bar color denotes slope direction: lighter for downregulation, darker for upregulation.

In whole blood, the strongest positive effects were observed in *Y_RNA* (0.39), *SBF2* (0.36), and *ABO* (0.31), and the latter regulated by rs495828 and rs507666. In the sigmoid colon, *SNX16* (0.40) and *ARL14EP*-*DT* (0.25; rs3858429, rs74485684) showed the highest activation, while *POLR2A* (rs12936464), (rs10256972), and *SEPTIN9* showed moderate negative slopes. In the ovary, *FAM86B3P* exhibited the highest positive slope (0.52; rs17603933), while *HEY2-AS1* and *LINC02523* showed negative values (~−0.50; rs2226158). In the ileum, *ENTR1P2* and *MRPS14* (0.34; rs34390425) were associated with increased expression, whereas *C7orf50* and *POLR2A* displayed more pronounced negative slopes (≤−0.38). These results are summarized in Figure 5, which graphically presents the regulatory effects per gene and tissue, and in Table 2, which details the variants associated with each gene.

### 3.4. Functional Annotation of eQTL-Regulated Genes in Molecular Signature Sets

To evaluate the biological relevance of the previously presented genes, including those most frequently regulated by eQTLs and those with the highest regulatory effect based on slope values, we performed functional annotation analyses using two curated resources: the MSigDB Hallmark Gene Sets and the Cancer Hallmarks Gene Set. Analyses were conducted separately for each tissue. This approach helped identify key biological processes potentially involved in the pathophysiology of endometriosis.

Genes such as *GATA4*, *CLDN23*, *MICB*, *MFHAS1*, and *BMPR2* were enriched in multiple signatures including estrogen response, coagulation, DNA repair, tumor-promoting inflammation, immune evasion, angiogenesis, sustained proliferative signaling, resistance to cell death, and metastasis. *GATA4* and *CLDN23*, in particular, were consistently associated with tissue invasion, proliferative signaling, and inflammatory response across up to five tissues. *MICB*, *HCP5*, and *C2* were mainly linked to immune-related pathways, while *MFHAS1* and *BMPR2* were associated with growth suppression, cell cycle regulation, and replicative immortality. Other genes such as *WNT4*, *F5*, and *SLC19A2* showed enrichment in specific processes including p53 signaling and complement activation.

**Figure 6:**
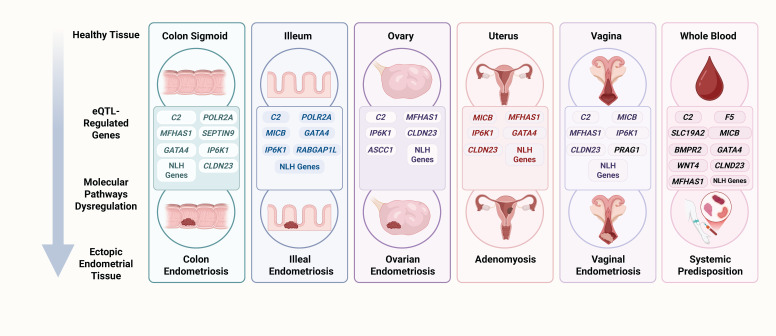
eQTL-regulated genes in endometriosis. In healthy tissue, genes modulated by eQTL variants may dysregulate molecular pathways and trigger pathogenic processes that promote the development of endometriosis. For example, in the sigmoid colon and ileum, the regulation of *C2* and *POLR2A* may impact complement/coagulation pathways and DNA repair mechanisms, generating a pro-inflammatory state with potential genomic instability (Table 3). In reproductive organs (uterus and ovary), eQTLs regulating *MICB*, *MFHAS1*, and *CLDN23* may alter estrogen response and heme metabolism, linking to tumor inflammation signatures, sustained proliferation, and the evasion of growth suppressors (Table 3 and Table 4). Likewise, the modulation of *GATA4* and *BMPR2* activates sustained angiogenesis and immune evasion pathways (Table 4), which may be essential for the vascularization and survival of ectopic implants. In the vagina, variants regulating *MFHAS1*, *CLDN23*, and *MICB-DT* affect epithelial barrier integrity and enhance local inflammatory signals and immune evasion (Table 3 and Table 4), facilitating the establishment of ectopic lesions in this tissue. Similarly, in peripheral blood, eQTLs regulating *MICB*, *HCP5*, and *F5* may promote the reprogramming of immune evasion and systemic angiogenesis (Table 4), suggesting an immunovascular component that could amplify pro-endometriotic signaling at distant sites. These examples demonstrate how, starting from an initially healthy tissue, eQTLs in key genes may orchestrate biological cascades that predispose to the implantation, proliferation, and persistence of endometrial tissue in ectopic locations. NLH refers to genes not linked to any hallmark (Table 3 and Table 4), created with Biorender.com, accessed on 6 July 2025.

Several frequently regulated genes, however, were not linked to any known signature in these databases. This group included *LINC00208*, *XKR6*, *USP4*, *C7ORF50*, *MICB*-*DT*, *LINC02949*, and *IP6K1*, many of which correspond to non-coding or poorly characterized transcripts. Detailed enrichment results by tissue are provided in Table 3 (MSigDB Hallmarks), Table 4 (Cancer Hallmarks), and Figure 6.

## 4. Discussion

In this study, we identified 465 genetic variants associated with endometriosis, distributed across all autosomal chromosomes and even the X chromosome. This widespread genomic distribution underscores the polygenic and complex nature of the disease, suggesting that multiple biological pathways contribute to disease risk. The most significantly associated variants in our analysis (e.g., rs10917151, rs56319427, rs72665317, rs11674184, rs1903068, rs71575922, rs13211170, rs17215781, rs11031005, and rs3858429) are in key genomic loci, primarily on chromosomes 1, 6, and 11, which include regions previously implicated in endometriosis. Several of these variants are consistent with findings from prior studies, reinforcing their role in disease susceptibility [15,16,17]. These studies have reported relevant loci on some of these chromosomes, which may suggest the presence of genomic regions enriched in regulatory elements or genes involved in reproductive and immunological processes.

Notably, the ten most significant variants highlight genomic regions with strong associations that may have relevant functional implications. Three of them (rs10917151, rs56319427, and rs72665317) are located on chromosome 1. The variant rs10917151, in addition to its association with endometriosis [10,18], has also been implicated in other gynecological conditions such as uterine fibroids [19], ovarian cancer [20] and leiomyomas [21]. Meanwhile, rs72665317 has also been associated with anovulatory infertility [22]. In contrast, rs56319427 has been exclusively linked to endometriosis [18]. Additionally, the variants rs11674184 and rs1903068, located on chromosomes 2 and 4, respectively, have so far only been associated with this condition [23].

Furthermore, among the variants located on chromosome 6, rs71575922 has been associated not only with endometriosis but also with uterine leiomyoma [8], while rs13211170 and rs17215781 have been linked exclusively to endometriosis. Regarding the variants located on chromosome 11, rs11031005 has been associated with both endometriosis [24] and polycystic ovary syndrome in women [25], whereas rs3858429 has only been associated with endometriosis [26].

In the functional annotation analysis of the variants identified in the GWAS we observed that the vast majority are located in non-coding regions, primarily intergenic, intronic, regulatory elements, and 3′ UTR sites. This pattern is consistent with findings in other complex diseases, where risk variants tend to influence gene regulation rather than protein structure [27,28,29]. Far from being mere accessory sequences, these regulatory elements act as control nodes within the genomic system. Their influence goes beyond simply turning genes on or off, they orchestrate when, where, and to what extent genes are expressed, shaping specific transcriptomic profiles depending on cellular context, physiological state, or pathological signals [30,31]. This dynamic modulation capacity positions regulatory elements as strategic components in the functional architecture of the genome. Altogether, these findings suggest that endometriosis is largely influenced by subtle changes in the expression of key genes, rather than by drastic mutations in protein-coding regions. Indeed, we identified a smaller subset of variants that are coding (such as missense or synonymous changes in exons), suggesting that some protein-coding genes may also contribute directly to disease risk when their sequences are altered, although these appear to be in the minority. For instance, somatic mutations in the *KRAS* gene have previously been reported in endometriosis [4]. These findings highlight the need for further functional studies to determine how both non-coding and coding variants alter cellular pathways related to the implantation and persistence of ectopic endometrial tissue.

A notable finding was the identification of specific genes that harbor a high number of variants associated with endometriosis. In particular, loci containing *CDKN2B-AS1*, *MFHAS1*, *WNT4*, and *SMAD3* emerged with multiple risk variants. The presence of independent signals within the same gene strongly suggests that these genomic regions harbor critical factors contributing to the disease. *CDKN2B-AS1* is a long non-coding RNA that modulates the expression of tumor suppressor genes such as *CDKN2A/B*, which are involved in cell cycle control and senescence [32]. Interestingly, this gene has been implicated in the development of ovarian endometriosis through its direct regulation of miRNAs that influence molecular pathways involved in cell proliferation and invasion [33]. Furthermore, it has also been described as a key marker in the promotion of malignancy in endometrial cancer [34]. The significant accumulation of variants at this locus observed in our analysis suggests a potential imbalance in the genetic regulatory network within ectopic endometrial tissue. Alterations in *CDKN2B-AS1* may foster a transcriptomic environment that evades normal cell cycle control, promoting the aberrant survival of endometrial cells outside the uterus. This dysregulation could represent a critical node in the pathophysiology of endometriosis, particularly in its more invasive forms, and supports the potential of *CDKN2B-AS1* not only as a genetic susceptibility marker but also as a potential therapeutic target in strategies aimed at modulating lncRNA activity.

Similarly, the consistent association of variants in *WNT4* reinforces its proposed role, as it is considered essential for the development of the reproductive tract and endometrial homeostasis [35]. Thus, genetic variants that may alter its expression or function could predispose to both eutopic and ectopic environments favorable for endometrial implantation [36]. *SMAD3*, on the other hand, has been shown to be upregulated in patients with endometriosis [37] suggesting that regulatory variants in this gene may influence the pathology and progression of the disease. Regarding *MFHAS1*, although it is a less characterized gene, a recent study identified *MFHAS1* as a potential shared genetic locus between endometriosis and asthma, suggesting its involvement in shared immuno-inflammatory mechanisms [38]. The accumulation of variants in *MFHAS1* may indicate a contribution of immune response or cellular signaling pathways in endometriosis, a hypothesis that warrants further investigation. Collectively, these genes emerge as important genetic nodes; future research focused on them may help elucidate their precise roles in the pathogenic cascade of endometriosis.

To connect genetic findings with specific cellular functions, we conducted an eQTL analysis on six tissues relevant to the pathophysiology of endometriosis: the sigmoid colon, ileum, ovary, uterus, vagina, and peripheral blood. This strategy enabled the identification of genes whose expression is modulated by variants previously associated with disease risk, providing a potential functional link between genotype and the cellular phenotypes involved in endometriosis.

Overall, we observed clear tissue specificity in gene regulation mediated by eQTL variants. Certain genes stood out due to their high number of regulatory variants, although their distribution varied significantly across tissues. In the sigmoid colon, the most regulated genes were *MFHAS1* (32 variants), *XKR6* (21), and *CLDN23* (20), all of which are involved in immune signaling, epithelial remodeling, and maintenance of mucosal barriers [38,39,40]. The accumulation of variants in genes related to intestinal homeostasis suggests that, although the colon is not a primary site of endometriotic implantation, it may represent a biological environment where specific variants enhance the activation of peripheral inflammatory responses, indirectly contributing to the systemic progression of the disease.

In the ileum, although fewer variants per gene were identified, *XKR6*, *IP6K1*, and *C2* stood out, suggesting the potential modulation of the mucosal immune system and local energy metabolism [40,41,42]. Considering that intestinal endometriosis can affect deep regions of the gastrointestinal tract [43], the regulation of these genes may contribute to the local inflammatory response or tissue remodeling in infiltrative lesions, facilitating their establishment or persistence within the intestinal environment.

In peripheral blood, the most regulated gene was *XKR6* (33 eQTLs), followed by *MICB-DT* and *HCP5* (25 each), as well as *MFHAS1* (18), all of which are known to have immunomodulatory functions [40,44,45,46]. These findings support the hypothesis of a systemic immune component in the pathophysiology of endometriosis [47,48].

In contrast, reproductive tissues (ovary, uterus, and vagina) exhibited distinct patterns. In the ovary, *MFHAS1* (17), *CLDN23* (13), and *USP4* (6) were the most regulated genes, all previously implicated in epithelial regulation, adhesion signaling, and tissue remodeling [39,49,50]. In the uterus, *MFHAS1*, *CLDN23*, *MICB-DT*, *HCP5*, and *LINC00208* were recurrently regulated, many of them shared with the ovary, although in different combinations. In the vagina, the most regulated genes again included *MFHAS1*, *CLDN23*, and *MICB-DT*, along with *PRAG1*, reflecting a mixed pattern of immunological and structural regulation [51]. This divergence in the most affected genes across tissues suggests that the functional impact of risk variants may be highly dependent on cellular context.

In addition to the number of regulatory variants per tissue, we also examined the magnitude of the regulatory effect using the average slope values. This allowed us to identify genes whose expression is significantly up- or downregulated by risk variants. This information provides a key functional dimension, as genes with marked positive or negative slopes may play decisive roles in the pathophysiology of endometriosis.

In the sigmoid colon and ileum, moderate negative effects were observed in genes such as *POLR2A* (−0.37) and *C7orf50* (−0.35). *POLR2A* is a major regulator of the expression of thousands of eukaryotic genes [52], and its inhibition has been shown to affect proliferation, survival, and tumorigenic capacity in colorectal cancer cells [53]. This suggests that reduced expression of *POLR2A* may interfere with the normal transcriptional response of the colonic epithelium to inflammatory or estrogenic stimuli, compromising mucosal integrity and potentially favoring ectopic colonization or the persistence of infiltrative lesions in the large intestine.

On the other hand, *C7orf50* has been proposed to have physiological relevance in intestinal tissue and possibly a key metabolic role in adipose tissue [54]. Its dysregulation in women carrying risk variants could disrupt the metabolic balance of intestinal mucosa, generating a permissive microenvironment for lesion persistence or infiltration.

The gene *SNX16* stood out for exhibiting a high positive slope (0.40) in the colon, while *MRPS14* (0.34) and *ENTR1P2*(0.34) showed similarly elevated values in the ileum. *SNX16* has previously been described as acting as an oncogene in hepatocellular carcinoma, promoting tumor aggressiveness through the EGFR–AKT pathway, and its inhibition has been proposed to offer therapeutic benefits by blocking tumor cell proliferation and invasion [55]. In addition, *MRPS14*, as part of the mitochondrial ribosome, has been reported to be significantly overexpressed in hepatocellular carcinoma, suggesting a potential role in tumor metabolic reprogramming and as a diagnostic biomarker [56]. In contrast, *ENTR1P2* does not yet have a clearly defined molecular function or known associations with disease or pathophysiological processes. However, the magnitude of its positive regulation in the ileum observed in our analysis raises the possibility that this transcript may represent a previously uncharacterized regulatory element, whose expression could be responsive to local inflammatory or metabolic signals associated with the progression of intestinal endometriotic lesions. Collectively, these findings support the notion that risk variants may alter the expression of genes involved in proliferation pathways, mitochondrial metabolism, or epithelial responses, contributing to the formation of a microenvironment that favors the persistence of infiltrative lesions in the gastrointestinal tract.

The ovary exhibited greater heterogeneity. Markedly negative slopes were observed in *LINC02523* and *HEY2-AS1* (−0.50), as well as in *NGF-AS1* (−0.47) and *LINC02949* (−0.49), all non-coding RNAs that have not been previously reported in any disease context. Our findings suggest that these transcripts may modulate regulatory signaling networks, potentially related to hormonal pathways, cell adhesion processes, or epithelial differentiation. Given that the ovarian microenvironment in women with endometriosis is characterized by an imbalance between proliferation and apoptosis, as well as altered hormonal responses [2,57], it is plausible that the dysregulation of these lncRNAs represents an additional epigenetic mechanism that contributes to chronic inflammation or ovarian dysfunction observed in this disease. Although speculative, this hypothesis reinforces the need to investigate the functional role of long non-coding RNAs in hormone-dependent tissues affected by endometriosis. On the other hand, *FAM86B3P* showed the highest positive slope across the entire dataset (0.52), suggesting an active role in ovarian processes not yet fully understood, possibly involving proliferation or epithelial regulation.

In the uterus, *MICB*, *MICB-DT*, and *HCP5* showed similar positive slopes (~0.61–0.62), while *GATA4* and *LINC00208* exhibited strongly negative effects (−0.51). *MICB* expression has been reported to increase in response to oxidative stress [58] and its overexpression may be associated with favorable prognosis in colorectal cancer [59]. *MICB-DT* is a non-coding RNA thought to regulate *MICB* expression [60], suggesting that the *MICB/MICB-DT* regulatory axis may play a role in maintaining local immune balance. Additionally, *HCP5*, another immunoregulatory lncRNA, showed a significant positive slope. While the elevated expression of *HCP5* has been linked to poorer survival in several cancers [61], in the context of endometriosis its overexpression could reflect persistent inflammatory signaling or local immune alterations that favor chronicity of ectopic tissue. Conversely, *GATA4*, a key transcription factor in reproductive organ development [62], was negatively regulated, possibly indicating impaired cellular differentiation and reduced capacity of the endometrium to respond to hormonal cues. The downregulation of *LINC00208*, a lncRNA with still poorly understood function, also suggests that non-coding elements may be modulating critical pathways in the uterus affected by endometriosis, disrupting its transcriptomic functionality. Altogether, these findings point toward a complex regulatory network (comprising both coding and non-coding genes) that may contribute to the establishment of a pro-inflammatory, immunologically altered, and hormonally dysfunctional environment.

In the vaginal tissue, the strongest positive effects of the entire study were observed, particularly in *MICB* (0.92), *MICB-DT* (0.80), and *HCP5* (0.80), suggesting robust activation of local regulatory axes, similar to what was observed in the ovary. In contrast, *C7orf50* (−0.53) and *CLDN23* (−0.51) showed strong repression, possibly indicating compromised epithelial barrier integrity and impaired cell adhesion mechanisms.

Finally, in peripheral blood, moderate positive effects were identified in *Y_RNA* (0.39), *SBF2* (0.36), and *ABO* (0.31). *Y_RNA* has been described as a readily accessible biomarker, detectable in serum, and potentially useful as a prognostic marker in various cancers due to its involvement in several cellular processes [63]. Although the role of *SBF2* remains poorly characterized, it has been associated with taxane-induced peripheral neuropathy [64]. While *ABO* has not been directly linked to endometrial cancer [65], its differential expression may reflect systemic inflammatory or immune alterations. These findings raise the possibility of exploring transcriptomic profiles in peripheral blood as a non-invasive strategy for monitoring endometriosis and its extrauterine manifestations.

On the other hand, among the negatively regulated genes, *FAM117B* and *BMPR2* stood out (−0.37 to −0.41). *FAM117B* has been implicated in the promotion of gastric cancer, particularly through pathways related to cell proliferation and resistance to apoptosis [66]. Regarding *BMPR2*, loss-of-function mutations have been associated with increased susceptibility to proliferative intestinal disorders such as colorectal polyps [67].

Finally, both the genes with the highest number of regulatory variants per tissue and those with the most pronounced expression effects represent key candidates for understanding the tissue-specific mechanisms involved in endometriosis. The results of our study suggest that the recurrence of certain genes across multiple tissues may reflect common regulatory nodes, while the specificity of others could indicate localized pathways of dysfunction. In this context, the identified eQTL variants may not only modulate critical biological functions but could also serve as indirect molecular markers of altered gene activity potentially associated with the onset of endometriosis or even with its clinical phenotypes. These findings open the door to future experimental validations, where the characterization of these genes and their regulatory variants may contribute to the development of diagnostic or prognostic biomarkers, specific to tissue or disease subtype. Based on these findings, we performed a functional assignment analysis using widely adopted gene collections, including the MSigDB Hallmark Gene Sets and a representative gene set reflecting the Cancer Hallmarks. This approach enabled us to contextualize the multiple genetic signals in terms of known biological pathways and cellular processes involving our candidate genes. The analysis was performed by tissue, considering both the genes with the highest number of eQTL variants and those with the most representative slope values.

In the sigmoid colon, we identified a marked activation of immune and tissue repair pathways. According to MSigDB, genes such as *C2*, *CLDN23*, and *POLR2A* were involved in gene signatures including allograft rejection, coagulation, complement, and DNA repair, suggesting a pro-inflammatory epithelial microenvironment with potential genomic instability processes that have been extensively reviewed and proposed as mediators of endometriosis [68], as well as potential promoters of carcinogenic progression [69,70]. In parallel, based on the Cancer Hallmarks Gene Set, these same genes were associated with processes such as evading immune destruction, tumor-promoting inflammation, sustained angiogenesis, and tissue invasion and metastasis, particularly through the actions of *CLDN23* and *GATA4*. Although the sigmoid colon is not a classical site of endometriotic implantation, these findings may reflect systemic or peripheral alterations that modulate immune responses and mucosal integrity, thereby contributing to the overall inflammatory context of the disease.

Our findings in the ileum revealed a potential interaction between mucosal immunity and cellular signaling associated with proliferative and immunometabolic processes. According to MSigDB, genes such as *C2*, *MICB*, *RABGAP1L*, and *IP6K1* were linked to signatures including evading immune destruction, IL2–STAT5 signaling, KRAS signaling Up, and notably, spermatogenesis. From the Cancer Hallmarks perspective, *GATA4*, *C2*, *MICB*, and *CLDN23* were implicated in evading immune destruction and tissue invasion, suggesting that even within intestinal tissues, relevant molecular signals associated with the pathophysiology of endometriosis may be present.

In particular, the activation of the KRAS pathway, a key regulator of cell proliferation and inflammation, reinforces the notion that certain oncogenic signaling routes may be involved in the onset and progression of endometriotic lesions, as previously reported [71,72]. Likewise, the dysregulation of the IL2–STAT5 pathway, known for its role in T cell homeostasis and immune response, has been implicated in endometrial cancer and may reflect an imbalance in local or systemic immune tolerance in women with endometriosis [73]. A particularly intriguing finding was the association with the spermatogenesis signature, typically linked to male spermatogenesis, but which in this context may indicate shared pathways or relevant processes in extra-gonadal tissues. Some proteins associated with this signature, such as *IP6K1*, have been implicated in cell signaling and energy metabolism [74], functions that may be relevant in ectopic endometrial cells or in local immune cells.

Altogether, these results support the existence of a complex network of immunometabolic dysfunction in the ileum, potentially modulated by the interplay between proliferative pathways, immune evasion mechanisms, and epigenetic signals. Understanding this network may broaden our insight into the systemic and multifocal nature of endometriosis.

In the ovary, (a tissue commonly affected by endometriosis) the prominent involvement of *MFHAS1*, *CLDN23*, *USP4*, and *GATA4* was identified, all of which are regulated by eQTLs. Functional analysis revealed associations with key signatures from both MSigDB and the Cancer Hallmarks collections. In MSigDB, these genes were linked to biological processes such as estrogen response, tumor-promoting inflammation, heme metabolism, and DNA repair, all of which are central mechanisms in the progression of endometriosis. For instance, the estrogen response is a fundamental axis in the hormonal regulation of ectopic implants [75,76], while tumor-promoting inflammation may facilitate local proliferation and immune evasion [68].

The association with heme metabolism supports hypotheses suggesting that cyclic blood accumulation within endometriotic lesions may lead to free iron toxicity, the generation of reactive oxygen species, and oxidative stress, ultimately promoting cellular damage, chronic inflammation, and potentially contributing to the persistence or transformation of ectopic tissue [77]. This finding suggests that dysregulation of heme metabolism in the ovary may play a significant pathogenic role.

From the Cancer Hallmarks perspective, these genes were associated with sustained angiogenesis, proliferative signaling, and tissue invasion; processes highly relevant for the implantation, vascularization, and expansion of endometrial tissue outside the uterus [78,79]. Sustained angiogenesis may support the establishment and nourishment of ectopic foci, while proliferative signaling and tissue invasion may facilitate infiltration into the ovarian stroma. Moreover, the association with DNA repair pathways indicates that impaired genomic maintenance may contribute to increased genomic instability in affected tissues, favoring the persistence of lesions.

Altogether, these findings reinforce the notion that ovarian endometriosis is not solely driven by hormonal stimuli but involves a complex molecular environment where genetically regulated processes (such as proliferation, inflammation, epithelial remodeling, and invasion) coexist, potentially modulated through eQTL mechanisms.

In the eutopic endometrium, altered transcriptomic profiles were identified that may predispose to early events of adhesion, invasion, and apoptotic evasion, thus promoting the development of endometriosis. Genes such as *BMPR2*, *MFHAS1*, and *CLDN23* were associated in MSigDB with pathways including TGF-β signaling, growth suppression, tissue invasion, and resistance to cell death, while in the Cancer Hallmarks set, genes such as *GATA4* and *CLDN23* were linked to evading growth suppressors, angiogenesis, and tissue invasion.

The activation of these signatures suggests that the endometrium in women with endometriosis may exhibit a pro-invasive molecular phenotype, resistant to cell death signals and with reduced proliferative restraint, even prior to the establishment of ectopic implants. Furthermore, the activation of angiogenic and tissue remodeling pathways reinforces the endometrium’s potential to generate a favorable environment for ectopic progression.

These findings support the notion that eutopic endometrium in endometriosis is not functionally normal, but rather exhibits early regulatory alterations [2,80], possibly driven by regulatory variants, which may play a key role in the pathophysiology of the disease. Although the vagina is an uncommon site for endometriosis, genes such as *CLDN23*, *MFHAS1*, and *PRAG1* showed notable functional associations. According to MSigDB, these genes were linked to tissue invasion, proliferative signaling, and evading immune destruction, while in the Cancer Hallmarks dataset, they were associated with tissue invasion and metastasis and evading immune destruction. These findings suggest that, even in atypical sites, molecular programs that promote adhesion, immune evasion, and local invasion may be activated. In particular, the presence of these molecular signatures reinforces the possibility that an altered epithelial environment could facilitate the colonization and persistence of endometriotic implants in this region.

In whole blood, the differential expression of genes regulated by eQTLs and linked to systemic inflammatory processes was observed. In MSigDB, genes such as *MICB*, *BMPR2*, *SLC19A2*, and *HCP5* were associated with key signatures including immune evasion, P53 pathway, angiogenesis, and resistance to cell death. From the Cancer Hallmarks perspective, genes such as *BMPR2*, *MICB*, *CLDN23*, and *WNT4* were linked to critical processes such as Replicative immortality, resistance to cell death, evading immune destruction, and angiogenesis.

The expression of these genes in blood supports the concept that endometriosis is not solely a localized disease but involves an active systemic immunological and vascular component [81,82]. Moreover, their detectability in peripheral blood highlights their potential value as non-invasive biomarkers for diagnosis or clinical monitoring.

Lastly, a key finding was the considerable proportion of genes that were not associated with any functional signature in the analyzed databases, including *LINC00208*, *XKR6*, *USP4*, *C7ORF50*, *MICB-DT*, and *LINC02949*, among others. Many of these correspond to non-coding or poorly characterized transcripts, highlighting important gaps in current functional annotation. These genes may be involved in biological processes not yet incorporated into canonical pathways, representing new opportunities to investigate novel mechanisms in the pathophysiology of endometriosis.

Although our analysis focused on identifying the eQTL effects of variants previously associated with endometriosis, it did not explore in depth the biological mechanisms through which these variants may exert their regulatory functions. Future studies analyzing specific or promising variants in endometriosis, especially within population contexts, may benefit from incorporating complementary computational tools to better characterize how such variants, particularly those located in non-coding regions, contribute to gene regulation and disease risk. In addition, while we focused on identifying the regulatory effects of known risk variants, future work applying statistical colocalization approaches could help confirm whether these effects reflect shared causal signals. Another limitation of this study is that the eQTL data from GTEx are derived from bulk tissue samples, which represent mixtures of different cell types. As a result, the observed regulatory effects reflect average expression changes across all cell populations within each tissue, potentially masking cell-type-specific signals. Future studies incorporating single-cell transcriptomic and eQTL data could provide more precise insights into the cell-type-specific regulatory mechanisms relevant to endometriosis. Overall, our findings reinforce the complex and tissue-specific nature of gene regulation in endometriosis and suggest that integrating eQTL variants with functional signatures may help uncover new molecular mechanisms and potential therapeutic targets.

## 5. Conclusions

Our results support the notion that endometriosis has a complex genetic architecture, driven primarily by non-coding variants that regulate the expression of key genes in disease-relevant tissues. By integrating GWAS, eQTL, and functional analyses, we identified molecular mechanisms linked to proliferation, immune evasion, epithelial remodeling, and apoptosis resistance. While some genes had been previously associated with the disease, our findings expand their functional context and highlight novel, underexplored candidates, including non-coding transcripts. These insights pave the way for future studies aimed at validating regulatory variants as potential biomarkers or therapeutic targets in endometriosis.

## Figures and Tables

**Table 1:** Distribution of GWAS variants associated with endometriosis across chromosomes.

Chromosome	No. of Variants	Top Significant Variant(s) (rsID) *	*p*-Value(s)
1	42	rs10917151, rs56319427, rs72665317	5 × 10^−44^, 4 × 10^−41^, 5 × 10^−34^
2	38	rs11674184	3 × 10^−26^
3	21		
4	17	rs1903068	7 × 10^−27^
5	19		
6	43	rs71575922, rs13211170, rs17215781	1 × 10^−31^, 6 × 10^−30^, 9 × 10^−27^
7	25		
8	66		
9	37		
10	33		
11	16	rs11031005, rs3858429	2 × 10^−32^, 6 × 10^−32^
12	28		
13	9		
14	9		
15	16		
16	1		
17	15		
18	6		
19	11		
20	4		
21	4		
22	1		
X	4		

* Variants ranked among the top 10 most statistically significant genome-wide associations (based on *p*-values), regardless of chromosome.

**Table 2:** Top 10 eQTL-regulated genes per tissue and their associated variants.

Tissue	Mapped Genes	Slope	Variants	Tissue	Mapped Genes	Slope	Variants
Colon Sigmoid	*SNX16*	0.4	rs114323125	Uterus	*HCP5*	0.62	rs2534685, rs2534687, rs2894221
Colon Sigmoid	*POLR2A*	−0.37	rs12936464	Uterus	*MICB-DT*	0.62	rs2534685, rs2534687, rs2894221
Colon Sigmoid	*C7orf50*	−0.35	rs10256972	Uterus	*MICB*	0.61	rs2516408
Colon Sigmoid	*SEPTIN9*	−0.33	rs98229	Uterus	*GATA4*	−0.51	rs13248109, rs13250871, rs4840573
Colon Sigmoid	*PPP1R3B-DT*	−0.26	rs1458942	Uterus	*LINC00208*	−0.51	rs13248109, rs13250871, rs4840573
Colon Sigmoid	*GATA4*	−0.25	rs13248109, rs13250871, rs4840573	Uterus	*NGF-AS1*	−0.46	rs7544256
Colon Sigmoid	*LINC00208*	−0.25	rs13248109, rs13250871, rs4840573	Uterus	*XKR6*	−0.43	rs10109025, rs11250097, rs11250098
Colon Sigmoid	*ARL14EP-DT*	0.25	rs11031005, rs3858429, rs74485684	Uterus	*FAM167A*	−0.41	rs12156009
Colon Sigmoid	*RPL35P2*	−0.25	rs112495680	Uterus	*FAM167A-AS1*	−0.41	rs12156009
Colon Sigmoid	*SMIM29*	−0.25	rs112495680	Uterus	*FAM117B*	−0.33	rs72928925
Ileum	*C7orf50*	−0.42	rs10256972	Vagina	*MICB*	0.92	rs2516408
Ileum	*POLR2A*	−0.38	rs12936464	Vagina	*HCP5*	0.8	rs2534685, rs2534687, rs2894221
Ileum	*ENTR1P2*	0.34	rs34390425	Vagina	*MICB-DT*	0.8	rs2534685, rs2534687, rs2894221
Ileum	*MRPS14*	0.34	rs34390425	Vagina	*C7orf50*	−0.53	rs10256972
Ileum	*MICB*	−0.33	rs2516408	Vagina	*CLDN23*	−0.51	rs519019, rs572366, rs693109, rs7825636, rs7829975
Ileum	*HCP5*	−0.33	rs2534685	Vagina	*LINC02949*	0.48	rs2976950
Ileum	*MICB-DT*	−0.33	rs2534685	Vagina	*LINC02950*	0.47	rs7837587
Ileum	*RABGAP1L*	0.26	rs4480415	Vagina	*FAM86B3P*	−0.46	rs17603933, rs55852693
Ileum	*GATA4*	−0.21	rs13248109, rs13250871, rs4840573	Vagina	*C2*	0.43	rs644045
Ileum	*LINC00208*	−0.21	rs13248109, rs13250871, rs4840573	Vagina	*ARL14EP-DT*	0.42	rs3858429, rs74485684
Ovary	*FAM86B3P*	0.52	rs17603933	Whole Blood	*FAM117B*	−0.41	rs72928925
Ovary	*HEY2-AS1*	−0.5	rs2226158	Whole Blood	*Y_RNA*	0.39	rs495828
Ovary	*LINC02523*	−0.5	rs2226158	Whole Blood	*BMPR2*	−0.37	rs6435157
Ovary	*LINC02949*	−0.49	rs2976950	Whole Blood	*MTCO1P17*	−0.37	rs6435157
Ovary	*NGF-AS1*	−0.47	rs7544256	Whole Blood	*SBF2*	0.36	rs59479500
Ovary	*C7orf50*	−0.35	rs10256972	Whole Blood	*ABO*	0.31	rs495828, rs507666
Ovary	*WDR27*	0.28	rs12193197	Whole Blood	*F5*	−0.3	rs1894692
Ovary	*ASCC1*	−0.23	rs7073342	Whole Blood	*SLC19A2*	−0.3	rs1894692
Ovary	*LINC02950*	−0.13	rs7837587	Whole Blood	*C7orf50*	−0.29	rs10256972
Ovary	*USP4*	−0.11	rs6778080	Whole Blood	*SLC9A2*	0.29	rs72828033

**Table 3:** Association of eQTL-regulated genes with MSigDB Hallmark Gene Sets across six tissues.

Hallmark Gene Set	Colon Sigmoid	Ileum	Ovary	Uterus	Vagina	Whole Blood
Allograft Rejection	*C2*	*C2*	*C2*		*C2*	*C2*
Coagulation	*C2*	*C2*	*C2*		*C2*	*C2*
Complement	*C2*	*C2*	*C2*		*C2*	*F5*, *C2*
DNA Repair	*POLR2A*	*POLR2A*				
Estrogen Response Early		*MICB*		*MICB*	*MICB*	*SLC19A2*, *MICB*
Estrogen Response Late		*MICB*		*MICB*	*MICB*	*MICB*
Heme Metabolism	*MFHAS1*		*MFHAS1*	*MFHAS1*	*MFHAS1*	*MFHAS1*
Il2 Stat5 Signaling		*RABGAP1L*				*BMPR2*
Kras Signaling Up		*RABGAP1L*				
Mitotic Spindle	*SEPTIN9*					
Spermatogenesis	*IP6K1*	*IP6K1*	*IP6K1*	*IP6K1*	*IP6K1*	
Tgf Beta Signaling						*BMPR2*
P53 Pathway						*SLC19A2*
Not Linked to Hallmark	*LINC00208*, *XKR6*, *C7ORF50*, *USP4*, *SNX16*, *PPP1R3B-DT*, *RPL35P2*, *CLDN23*, *SMIM29*, *ARL14EP-DT*, *GATA4*, *HCP5*, *MICB-DT*	*LINC00208*, *XKR6*, *C7ORF50*, *CDK2AP1*, *USP4*, *MRPS14*, *ENTR1P2*, *HCP5*, *GATA4*, *MICB-DT*	*LINC02950*, *RN7SL178P*, * NGF-AS1*, *C7ORF50*, *LINC02949*, *USP4*, *FAM86B3P*, *LINC02523*, *ASCC1*, *WDR27*, *CLDN23*, * HEY2-AS1*, *HCP5*, * MICB-DT*	*LINC00208*, *RN7SL178P*, *XKR6*, * NGF-AS1*, *USP4*, *FAM167A*, *FAM167A-AS1*, *CLDN23*, *FAM117B*, *GATA4*, *HCP5*, *MICB-DT*	*LINC02950*, *RN7SL178P*, *XKR6*, *LINC02949*, *C7ORF50*, *USP4*, *FAM86B3P*, *ABO*, *CLDN23*, * ARL14EP-DT*, *PRAG1*, *HCP5*, *MICB-DT*	*LINC00208*, *XKR6*, *WNT4*, *C7ORF50*, *Y_RNA*, *ABO*, *MTCO1P17*, *SBF2*, *CLDN23*, *FAM117B*, *GATA4*, *SLC9A2*, *HCP5*, *MICB-DT*

**Table 4:** Association of eQTL-regulated genes with Cancer Hallmark categories across six tissues.

Cancer Hallmark	Colon Sigmoid	Ileum	Ovary	Uterus	Vagina	Whole Blood
Sustained Angiogenesis	*GATA4*	*GATA4*		*GATA4*		*BMPR2*, *GATA4*, *WNT4*
Tumor-Promoting Inflammation	*CLDN23*		*CLDN23*	*CLDN23*	*CLDN23*	*CLDN23*
Genome Instability			*ASCC1*			
Sustaining Proliferative Signaling	*GATA4*	*GATA4*		*GATA4*		*BMPR2*, *GATA4*, *WNT4*
Evading Immune Destruction	*C2*, *CLDN23*	*MICB*, *C2*	*C2*, *CLDN23*	*MICB*, *CLDN23*	*MICB*, *C2*, *CLDN23*	*F5*, *MICB*, *C2*, *CLDN23*
Replicative Immortality						*BMPR2*, *WNT4*
Resisting Cell Death						*BMPR2*
Evading Growth Suppressors	*MFHAS1*		*MFHAS1*	*MFHAS1*	*MFHAS1*	*BMPR2*, *MFHAS1*, *WNT4*
Reprogramming Energy Metabolism						
Tissue Invasion and Metastasis	*GATA4*, *CLDN23*	*GATA4*	*CLDN23*	*GATA4*, *CLDN23*	*PRAG1*, *CLDN23*	*BMPR2*, *GATA4*, *WNT4*, *CLDN23*
Not Linked to Cancer Hallmark	*LINC00208*, *SEPTIN9*, *XKR6*, *C7ORF50*, *USP4*, *POLR2A*, *IP6K1*, *SNX16*, * PPP1R3B-DT*, *RPL35P2*, *HCP5*, *SMIM29*, * ARL14EP-DT*, *MICB-DT*	*LINC00208*, *XKR6*, *C7ORF50*, *CDK2AP1*, *USP4*, *MRPS14*, *RABGAP1L*, *POLR2A*, *IP6K1*, *ENTR1P2*, *HCP5*, * MICB-DT*	*LINC02950*, *RN7SL178P*, *NGF-AS1*, *C7ORF50*, *LINC02949*, *USP4*, *FAM86B3P*, *IP6K1*, *LINC02523*, *WDR27*, *HCP5*, *HEY2-AS1*, *MICB-DT*	*LINC00208*, *RN7SL178P*, *XKR6*, * NGF-AS1*, *USP4*, *FAM167A*, *IP6K1*, *FAM167A-AS1*, *HCP5*, *FAM117B*, *MICB-DT*	*LINC02950*, *RN7SL178P*, *XKR6*, *LINC02949*, *C7ORF50*, *USP4*, *FAM86B3P*, *IP6K1*, *ABO*, *HCP5*, *ARL14EP-DT*, *MICB-DT*	*LINC00208*, *XKR6*, *C7ORF50*, *SLC19A2*, *ABO*, *MTCO1P17*, *SBF2*, *HCP5*, *FAM117B*, *Y_RNA*, *SLC9A2*, *MICB-DT*

## Data Availability

Data are contained within the article.
